# Supplementary material for: A dynamic and adaptive network of cytosolic interactions governs protein export by the T3SS injectisome
Source: Nat Commun. 2017 Jun 27;8:15940. doi: 10.1038/ncomms15940 (PMC5490264; doi:10.1038/ncomms15940)
Supplement: Supplementary Information [file ncomms15940-s1.pdf]

File name: Supplementary Information
Description: Supplementary Figures, Supplementary Tables and Supplementary References

File name: Supplementary Movie 1
Description: EGFP-YscQ localization time course with exposure of 100 ms / frame (frame rate 1/180 ms)

File name: Supplementary Movie 2
Description: EGFP-YscQ localization time course with exposure of 40 ms / frame (frame rate 1/120 ms)

File name: Supplementary Movie 3
Description: EGFP-YscQ localization time course with exposure of 15 ms / frame (frame rate 1/95 ms)

File name: Supplementary Movie 4
Description: EGFP-YscL localization time course with exposure of 100 ms / frame (frame rate 1/180 ms)

File name: Supplementary Movie 5
Description: EGFP-YscL localization time course with exposure of 40 ms / frame (frame rate 1/120 ms)

File name: Supplementary Movie 6
Description: EGFP-YscL localization time course with exposure of 15 ms / frame (frame rate 1/95 ms)

File name: Peer Review File
Description:

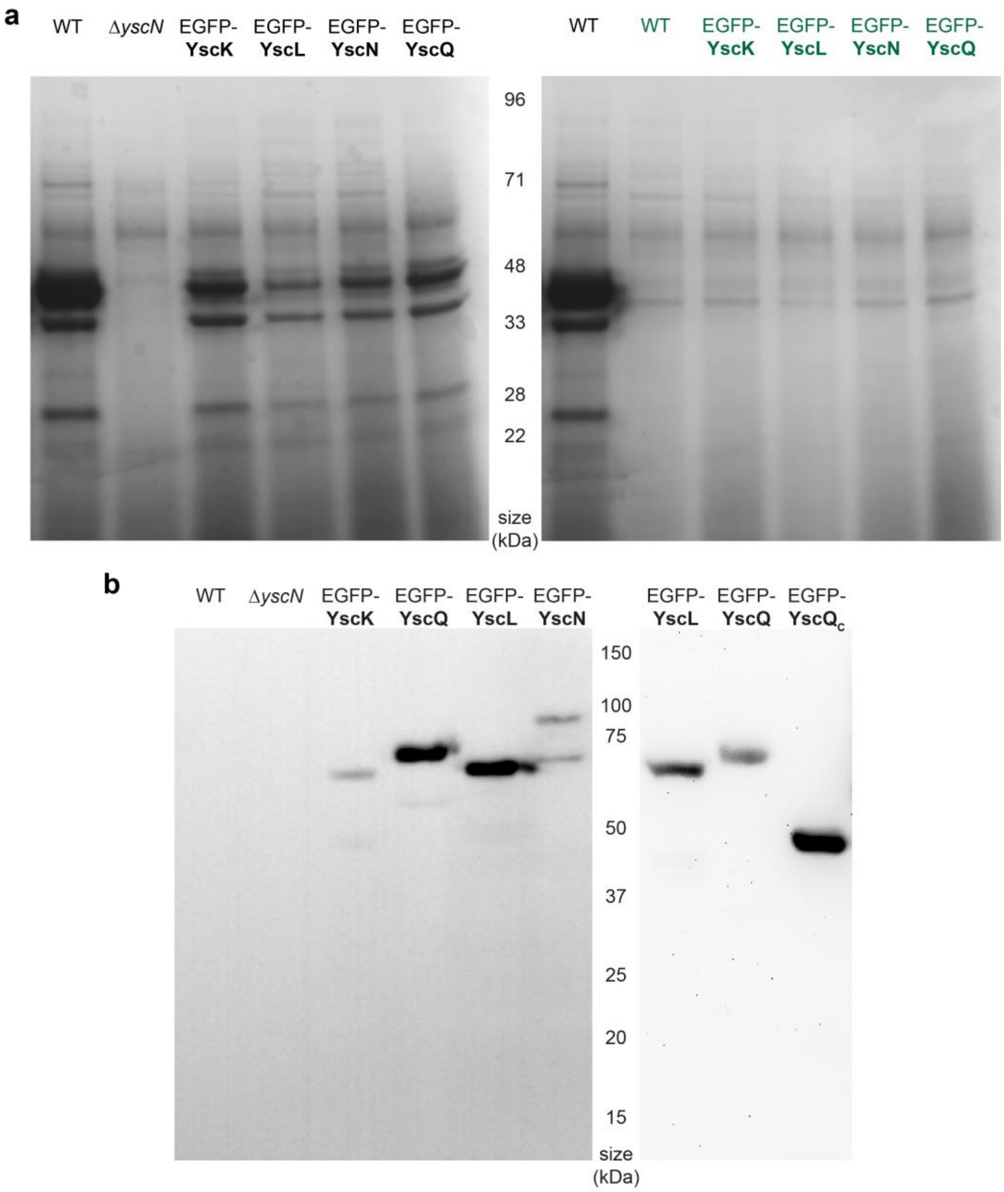

Supplementary Fig. 1: Secretion assay and immunoblot showing the functionality and stability of the used EGFP fusion proteins

a: Secretion profile (Coomassie staining of TCA-precipitated proteins in the culture supernatant after three hours of incubation at 37°C) of the indicated strains (wild-type, WT, and *ΔyscN* strains as positive and negative controls, and strains expressing EGFP fusion proteins as indicated from their native promoter on the pYV virulence plasmid) under secreting conditions (absence of Ca^{2+}, black) and non-secreting conditions (presence of Ca^{2+}, green).

b: Immunoblot anti-GFP of total cellular proteins in the indicated strains, as above. To assess the stability of the fusion proteins, untreated total cellular proteins were analysed using anti-GFP antibodies. For the fusions to YscK, YscQ, $YscQ_C$, and YscL, only minor (<5% intensity) degradation bands with molecular weights about 15-20 kDa below can be detected, similar to earlier results[1]. For EGFP-YscN, this degradation band is stronger. Importantly, no band corresponding to free EGFP (25-30 kDa) could be detected for any strain.

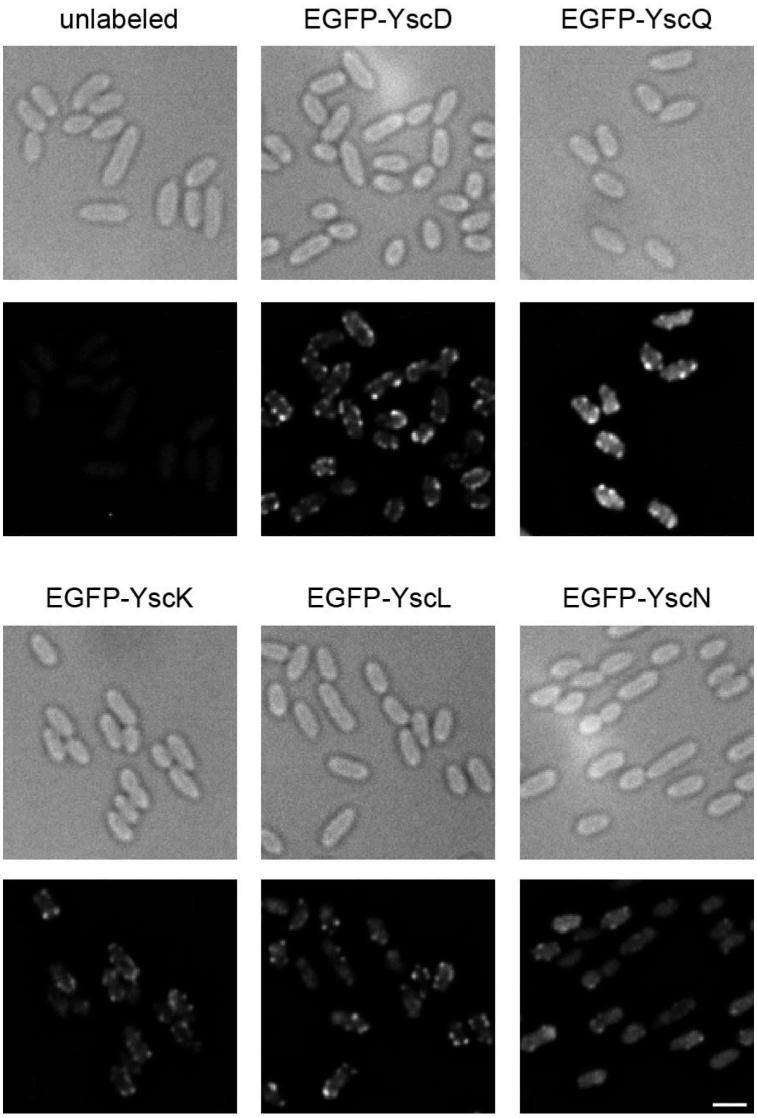

Supplementary Fig. 2: The overall localization of the soluble T3SS components does not change upon induction of secretion.

All soluble T3SS components localize in membrane foci under secreting conditions. Deconvoluted micrographs of bacteria expressing N-terminal EGFP fusions of the respective proteins under secreting conditions (see **Fig. 1b** for non-secreting conditions). The representative micrographs shown allow the comparison of fluorescence intensity. Each strain was imaged in three to four independent experiments yielding reproducible results. Scale bar, 2 µm.

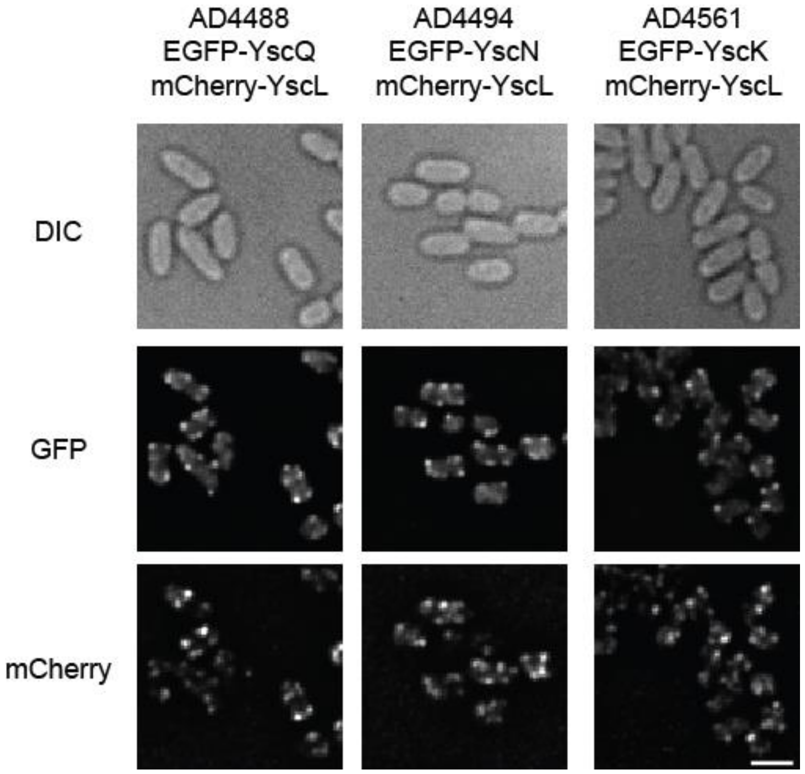

Supplementary Fig. 3: The soluble T3SS components colocalize with each other in double labelled bacteria.

Micrographs of EGFP and mCherry fluorescence in strains coexpressing two labelled soluble T3SS components under non-secreting conditions (see **Fig. 1c** for overlays). Each strain was imaged in three independent experiments yielding reproducible results. Scale bar, 2 µm.

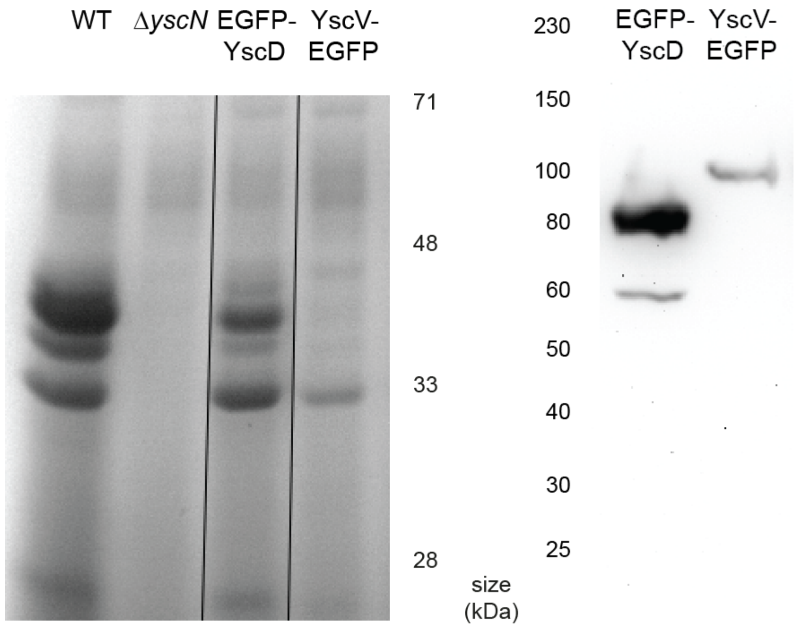

Supplementary Fig. 4: Stability and functionality of fluorescent fusions to the membrane-bound T3SS components YscD and YscV

Secretion profile (Coomassie staining of TCA-precipitated proteins in the culture supernatant after three hours of incubation at 37°C; left side) and immunoblot using an anti-GFP antibody (right side) of strains expressing EGFP-YscD or YscV-EGFP, as indicated. All samples were run and analysed on the same gel; vertical lines indicate omission of intermediate lanes.

Secretion of effectors is slightly decreased in the strain expressing EGFP-YscD, and strongly reduced in YscV-EGFP. Numbers and lines indicate protein size in kDa. The expected size of the fusion proteins is 75.2 kDa (EGFP-YscD) and 106.6 kDa (YscV-EGFP). The majority of fusion proteins runs at the expected level. A weak degradation band is visible for EGFP-YscD, likely representing degradation within YscD. Importantly, no band is visible in the range of GFP (25-30 kDa), indicating that the observed fluorescence is not due to cleavage of fluorophores.

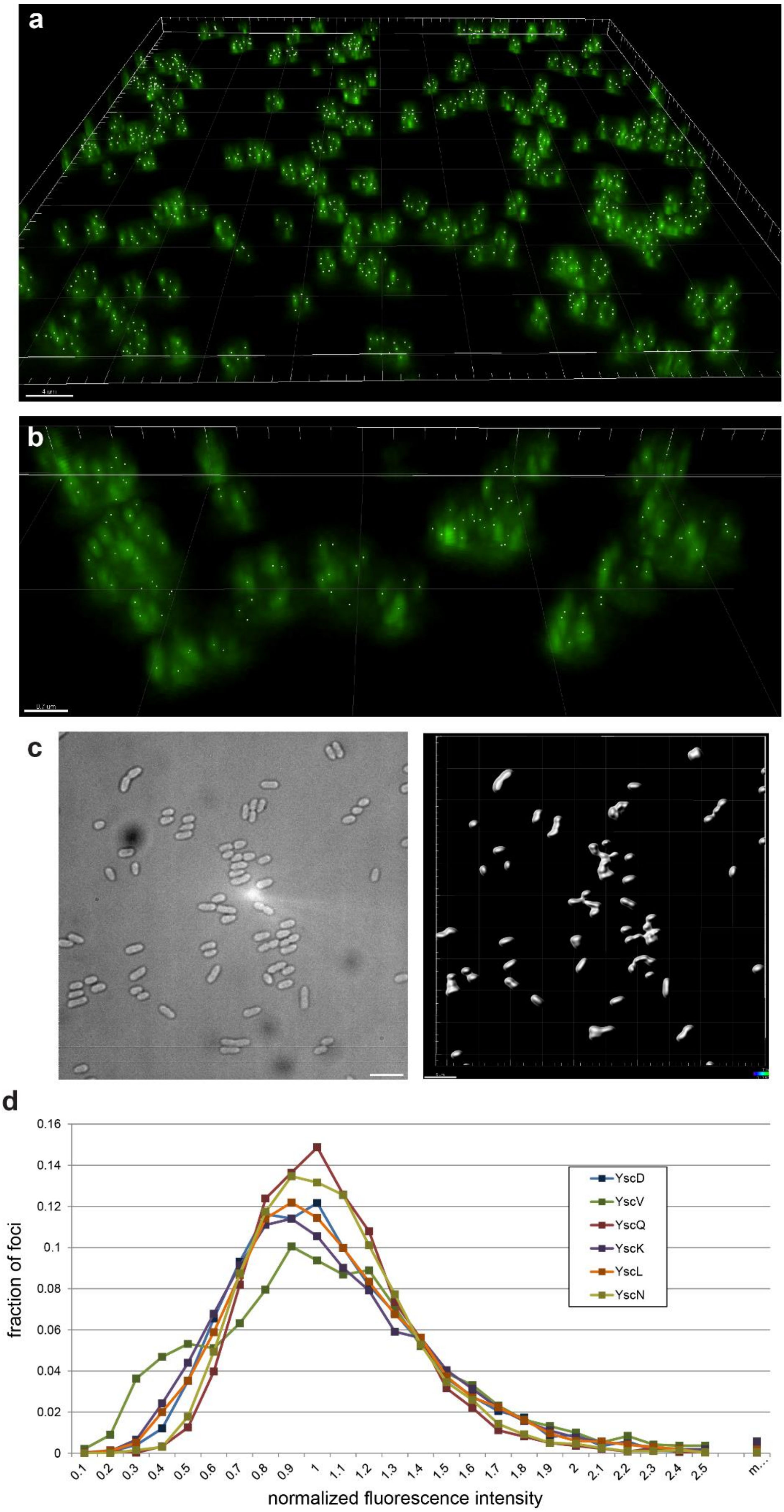
a
b
c
d
0.16
0.14
0.12
0.1
0.08
0.06
0.04
0.02
0
fraction of foci
0.1
0.2
0.3
0.4
0.5
0.6
0.7
0.8
0.9
1
1.1
1.2
1.3
1.4
1.5
1.6
1.7
1.8
1.9
2
2.1
2.2
2.3
2.4
2.5
m...
normalized fluorescence intensity
YscD
YscV
YscQ
YscK
YscL
YscN

Supplementary Fig. 5: Determination of the stoichiometry of the cytosolic complex

a, **b**: Overview and close-up view of three-dimensional fluorescence distribution and detection of fluorescent foci. Three-dimensional views of fluorescence distribution in strain ADTM4520 expressing EGFP-YscL, and centres of detected spots (green points). Height of z stack: 1.8 μm: scale bars: 4 μm (overview), 0.7 μm (close-up)

c: Representation of the region used to determine the average fluorescence background in an unlabelled strain (right) in comparison to the corresponding DIC image of the centre frame (left). Scale bars, 5 μm.

d: Normalized fluorescence intensity of detected foci in strains expressing N-terminal EGFP fusions of the respective proteins (with the exception of YscV-EGFP) under non-secreting conditions. Data as shown in **Fig. 1e**, average fluorescence intensities set to 1 for each strains. m…, more than 2.5x average intensity.

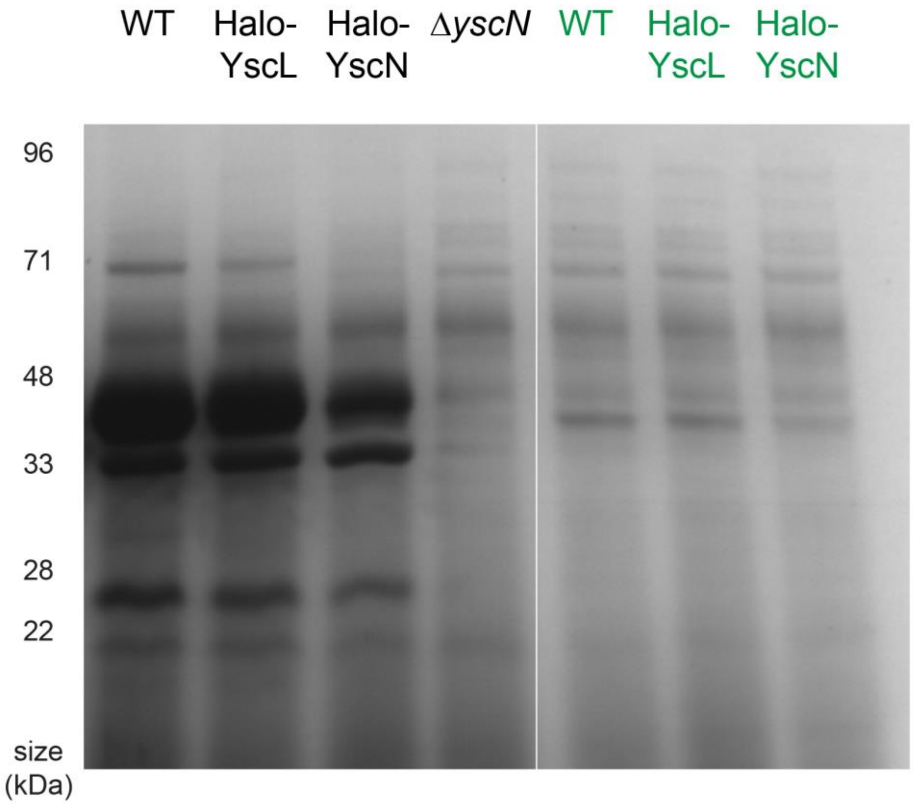

Supplementary Fig. 6: Secretion assay showing the functionality of the used Halo-tagged proteins

Secretion profile (Coomassie staining of TCA-precipitated proteins in the culture supernatant after three hours of incubation at 37°C) of the indicated strains under secreting conditions (absence of Ca^{2+}, black) and non-secreting conditions (presence of Ca^{2+}, green). All samples were analysed on the same gel, the white line indicates the omission of an intermediate lane.

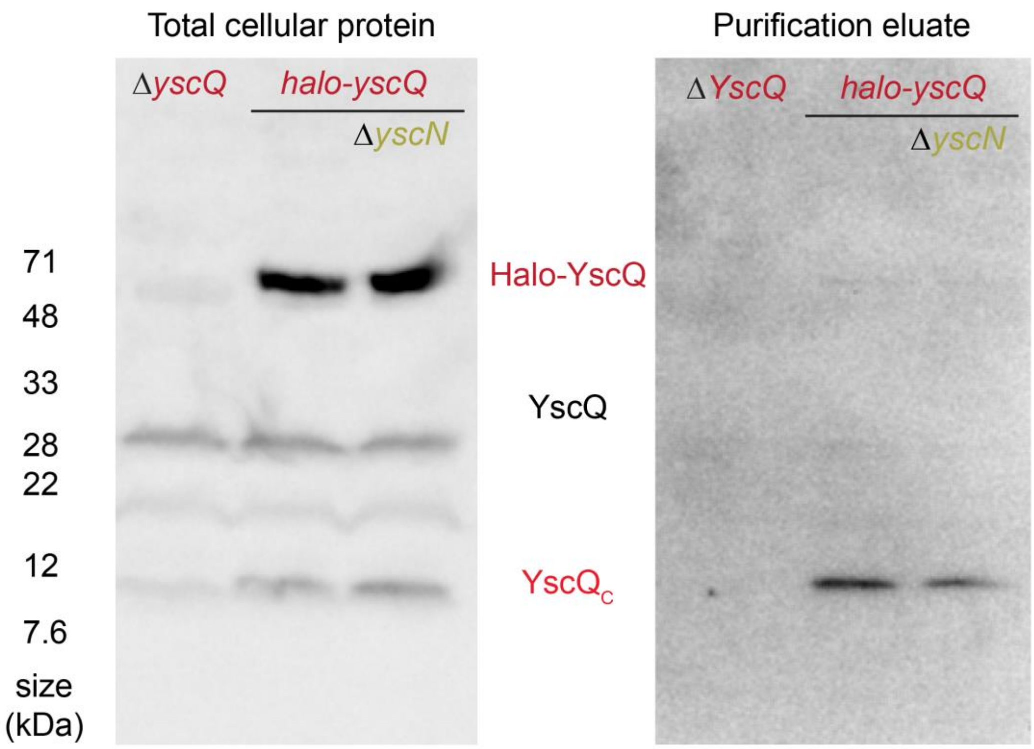

Supplementary Fig. 7: Expression of Halo-YscQ and YscQ$_C$ is not affected by deletion of YscN

Immunoblots using a polyclonal antibody directed against YscQ. Left side, total cellular protein from $2*10^8$ bacteria; right side, purification eluate from $2*10^9$ bacteria of the indicated strains. Standard protein sizes in kDa indicated on the left side, expected band sizes for Halo-YscQ (69.5 kDa), YscQ (34.4 kDa), and YscQ$_C$ (10,0 kDa) indicated in the centre.

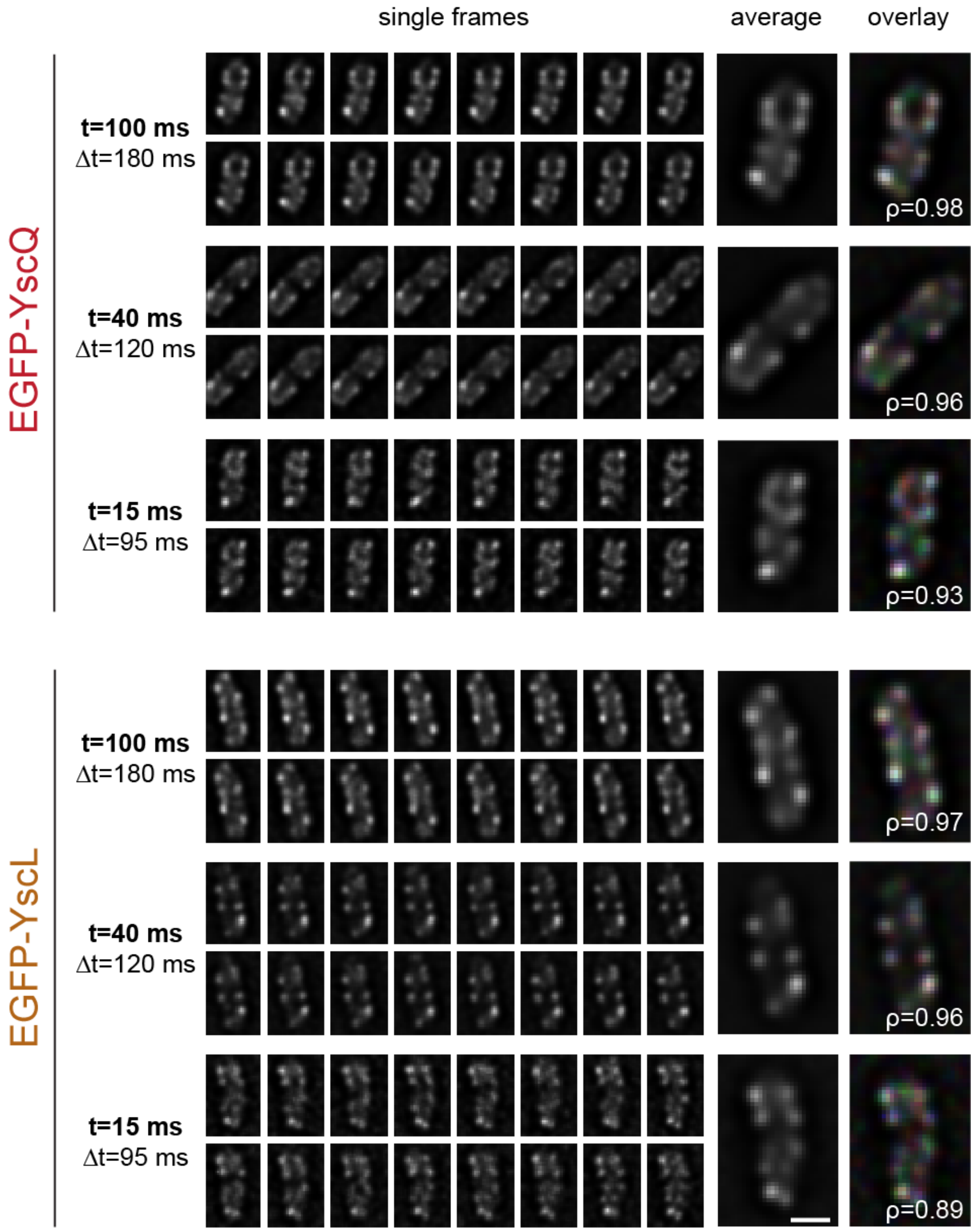

Supplementary Fig. 8: Highly time-resolved florescence microscopy indicates movement of complexes through the cytosol

Left: consecutive deconvoluted frames of time course microscopy visualizing EGFP-YscQ (top half) or EGFP-YscL (bottom half) under non-secreting conditions. Exposure time and frame rate (= exposure time + data transfer time) as indicated. Centre: Average of micrographs on the left. Right: overlay of three frames (red, green, blue channel) with Δt = 540-570 ms. White indicates overlapping fluorescence, whereas colours indicate dynamic fluorescence. ρ, Pearson correlation coefficient, average between the three images displayed in the overlay. Scale bar, 1 µm.

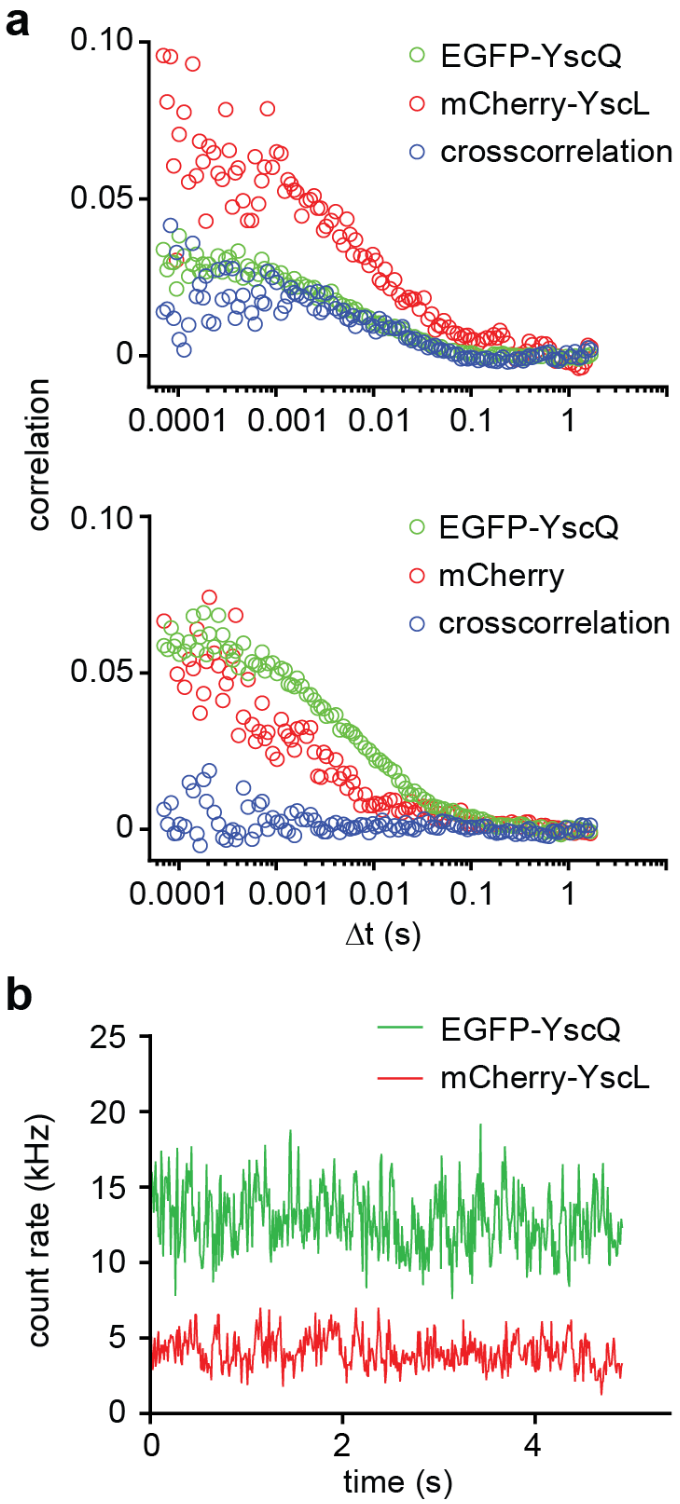

Supplementary Fig. 9: Cytosolic interactions between YscL and YscQ can be detected in live bacteria.

a: Cytosolic auto-correlation curves for given proteins (red and green circles), and cross-correlation (blue circles) for a strain expressing EGFP-YscQ and mCherry-YscL in a *ΔyscD* background (top) and the respective negative control (EGFP-YscQ, free mCherry, *ΔyscD*).

b: Representative intensity traces for EGFP-YscQ and mCherry-YscL.

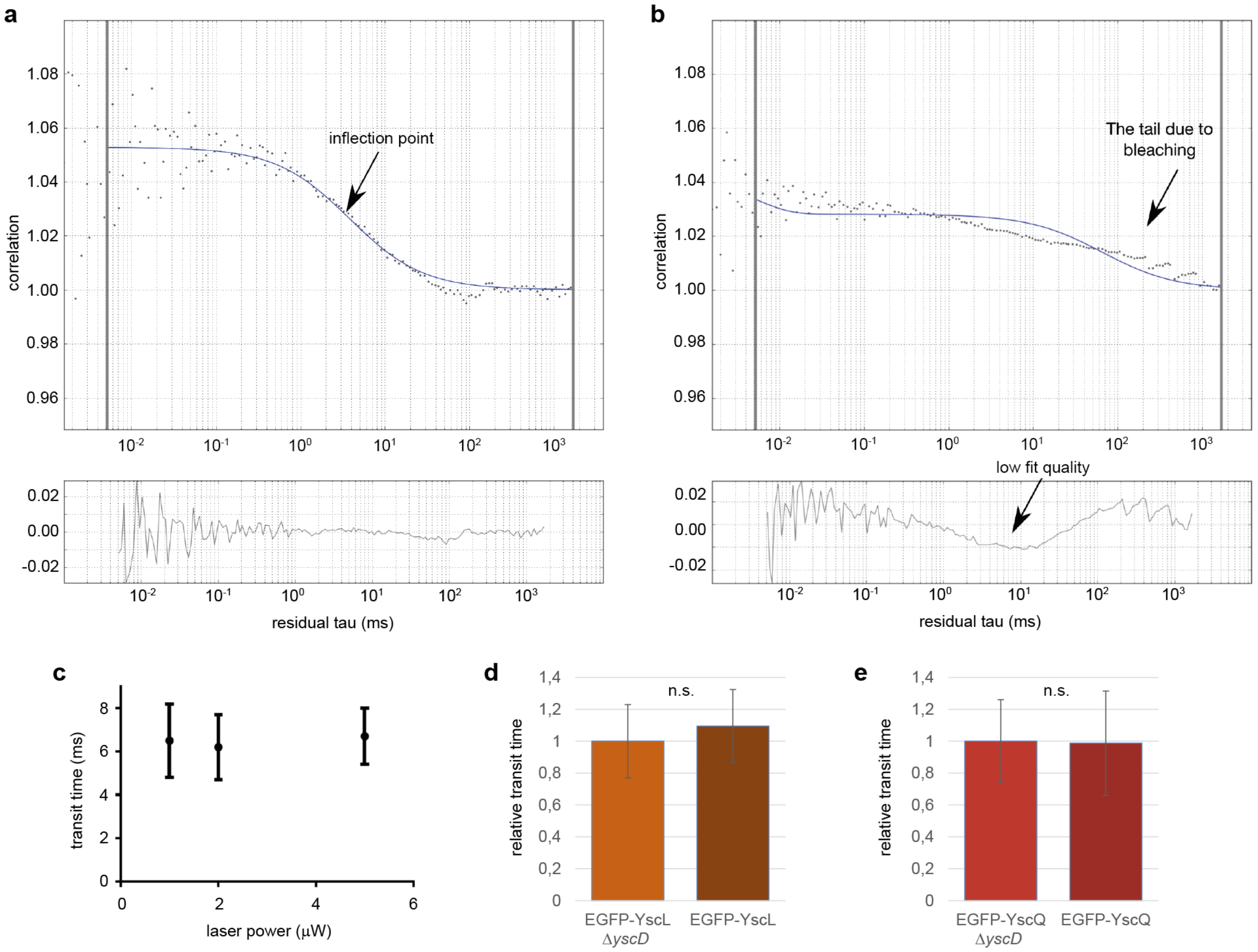

a
inflection point
correlation
residual tau (ms)
b
The tail due to bleaching
low fit quality
correlation
residual tau (ms)
c
transit time (ms)
laser power (µW)
d
n.s.
relative transit time
EGFP-YscL ΔyscD
EGFP-YscL
e
n.s.
relative transit time
EGFP-YscQ ΔyscD
EGFP-YscQ

Supplementary Fig. 10: Fluorescence correlation spectroscopy quality control and additional measurements

a, b: Snapshots from FoCuS-point software[2], showing autocorrelation data and fits for a representative FCS measurement (**a**) and a measurement, which was excluded due to bad fit quality caused by significant photobleaching (**b**).

c: Control experiment measuring the average transit time of EGFP-YscQ under non-secreting conditions, using different laser powers as indicated. Decreasing values for higher laser powers would be indicative of significant photobleaching[3]; this was not observed for our data. Error bars represent standard deviation from the mean of at least 10 curves.

d, e: The deletion of YscD does not influence the diffusion of EGFP-YscL or EGFP-YscQ. Transit times were measured in parallel for bacteria expressing EGFP-YscL (**d**) or EGFP-YscQ (**e**) expressed from their native genetic location, and otherwise WT (dark bars) or *ΔyscD* (light bars) (n=13-29, error bars represent standard deviation). The difference between WT strain and the respective *ΔyscD* strains is not statistically significant (n.s.) in a two-tailed homoscedastic t-test.

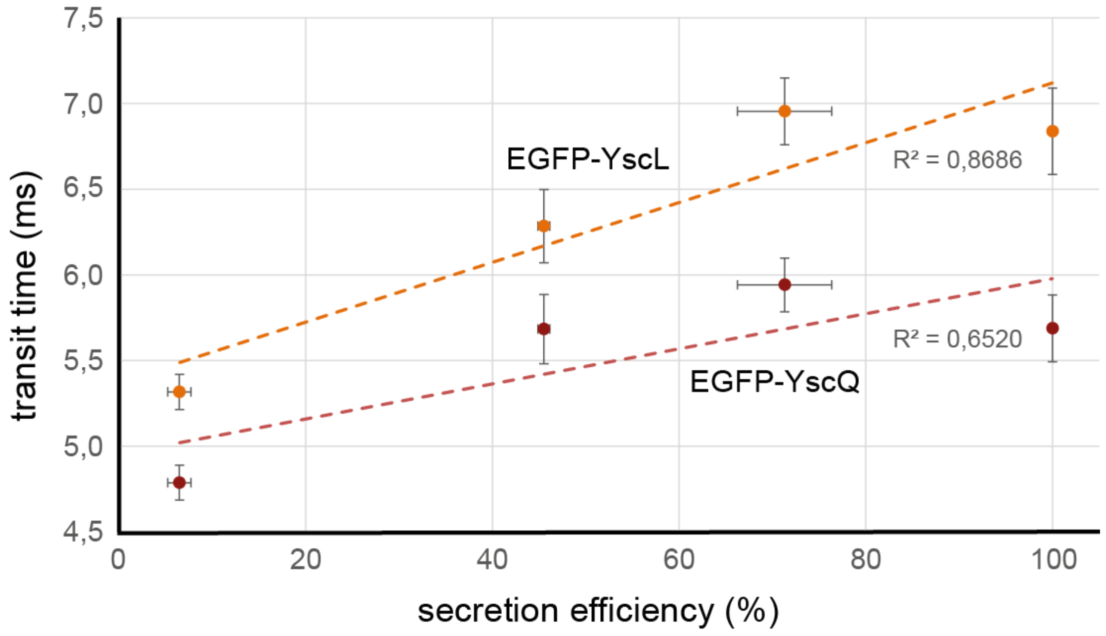

Supplementary Fig. 11: Correlation between secretion efficiency and mobility of the soluble T3SS components.

Correlation between secretion efficiency of a wild-type strain (x-axis, normalized to maximal efficiency), and transit times of EGFP-YscL (dark orange) and EGFP-YscQ (dark red) in the respective *ΔyscD* strains under the same conditions. Error bars represent standard errors of the mean value of the secretion efficiency (x axis, normalized to the maximal rate, n = 3) , and the transit time (y axis, n = 64-158).

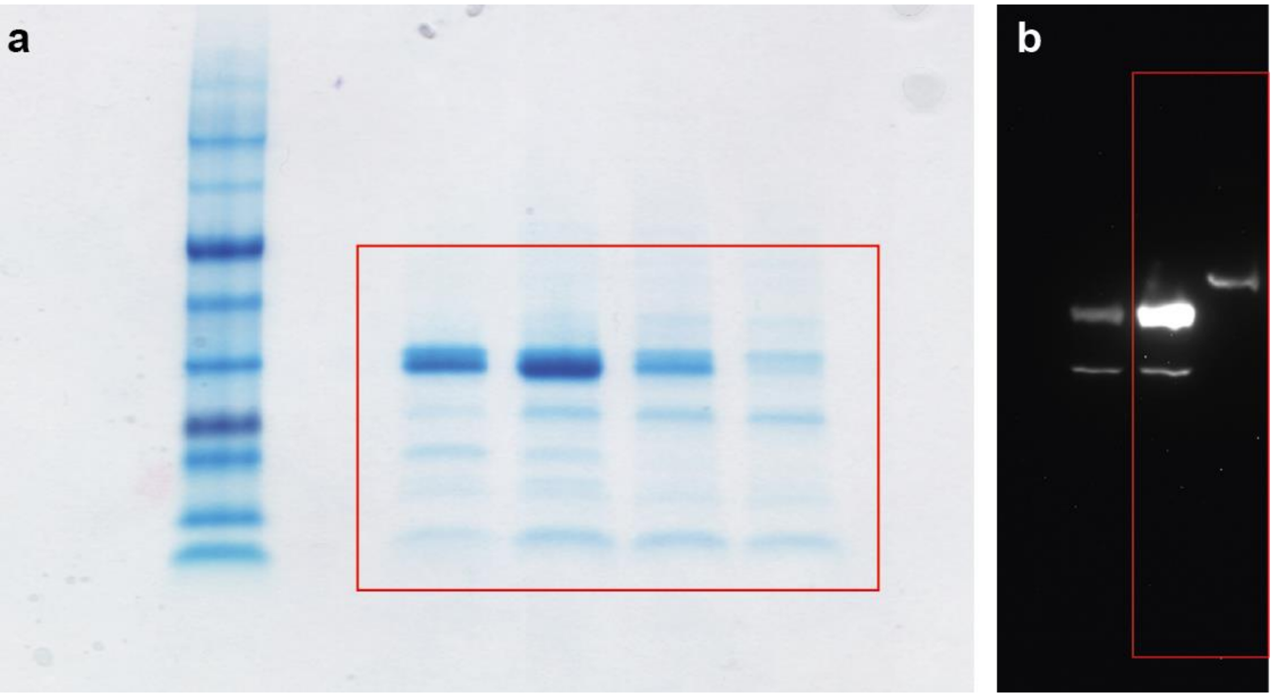

Supplementary Fig. 12: Uncropped versions of displayed gels and immunoblots

a: Uncropped version of the gel displayed in **Fig. 3d**.

b: Uncropped version of the immunoblot displayed in **Supplementary Fig. 4b**.

Red boxes indicate the approximate area displayed in the respective figures.

Supplementary Table 1

Homologous proteins and their function in various families of T3SS and the flagellum

General Sct names[4] and species-specific protein names are given for well-studied members of the respective T3SS families. -, no clear homologue present.

Functional name	Sct name	*Yersinia*	*Shigella*	*Salmonella SPI-1*	*Escherichia coli*	Flagellar homologue
Secretin	SctC	YscC	MxiD	InvG	EscC	-
Outer MS-ring protein	SctD	YscD	MxiG	PrgH	EscD	-
Inner MS-ring protein	SctJ	YscJ	MxiJ	PrgK	EscJ	FliF
Minor export apparatus protein	SctR	YscR	Spa24 (SpaP)	SpaP	EscR	FliP
Minor export apparatus protein	SctS	YscS	Spa9 (SpaQ)	SpaQ	EscS	FliQ
Minor export apparatus protein	SctT	YscT	Spa29 (SpaR)	SpaR	EscT	FliR
Export apparatus switch protein	SctU	YscU	Spa40 (SpaS)	SpaS	EscU	FlhB
Major export apparatus protein	SctV	YscV	MxiA	InvA	EscV	FlhA
Accessory cytosolic protein	SctK	YscK	MxiK	OrgA	-	-
C-ring protein	SctQ	YscQ	Spa33 (SpaO)	SpaO	EscQ	FliM + FliN
Regulator (stator)	SctL	YscL	MxiN	OrgB	EscL (Orf5)	FliH
ATPase	SctN	YscN	Spa47 (SpaL)	InvC	EscN	FliI
Stalk	SctO	YscO	Spa13 (SpaM)	InvI	Orf15	FliJ
Needle filament protein	SctF	YscF	MxiH	PrgI	EscF	-
Inner rod protein	SctI	YscI	MxiI	PrgJ	EscI (rOrf8)	-
Needle length regulator	SctP	YscP	Spa32 (SpaN)	InvJ	EscP (Orf16)	FliK
Hydrophilic translocator, needle tip protein		LcrV	IpaD	SipD	-	-
Hydrophobic translocator, pore protein		YopB	IpaB	SipB	EspD	-
Hydrophobic translocator, pore protein		YopD	IpaC	SipC	EspB	-
Pilotin		YscW	MxiM	InvH	-	-
Gatekeeper	SctW	YopN	MxiC	InvE	SepL	-

Supplementary Table 2

List of strains and plasmids used in this study

Relevant genotype of *Y. enterocolitica* strains. All strains are based on the multi-effector knock-out auxotrophic strain IML421*asd*, which is an E40-based strain.

Strain name	Relevant strain characteristics of the pYV virulence plasmid	References
IML421*asd*	pYV40 $yopO_{\Delta 2\text{-}427}$ $yopE_{21}$ $yopH_{\Delta 1\text{-}352}$ $yopM_{23}$ $yopP_{23}$ $yopT_{135}$ Δasd	[5]
AD4085	IML421 *egfp-yscQ*	[5]
AD4165	IML421 *yscV-egfp*	this study
AD4306	IML421 *egfp-yscD*	[1]
AD4392	IML421 *egfp-yscQ, ΔyscN*	this study
AD4393	IML421 *egfp-yscQ, mCherry-yscD*	this study
AD4396	IML421 egfp-$yscQ_{M218A}$	this study
AD4411	IML421 *egfp-yscQ ΔyscD*	this study
AD4419	IML412 *ΔyscQ*	[1]
AD4461	IML421 *halo-yscQ*	this study
AD4463	IML421 *halo-yscQ ΔyscN*	this study
AD4474	IML421 *egfp-yscK*	this study
AD4476	IML421 *egfp-yscK ΔyscD*	this study
AD4478	IML421 *egfp-yscK ΔyscQ*	this study
AD4483	IML421 *egfp-yscL ΔyscD*	this study
AD4484	IML421 *egfp-yscL ΔyscN*	this study
AD4485	IML421 *egfp-yscL ΔyscQ*	this study
AD4486	IML421 *egfp-yscN ΔyscD*	this study
AD4487	IML421 *egfp-yscN ΔyscL*	this study
AD4488	IML421 *egfp-yscQ mCherry-yscL*	this study
AD4489	IML421 *egfp-yscQ mCherry-yscL ΔyscD*	this study
AD4494	IML421 *egfp-yscN mCherry-yscL*	this study
AD4561	IML421 *egfp-yscK mCherry-yscL*	this study
ADMH4534	IML421 *halo-yscQ ΔyscD*	this study
ADTM4514	IML421 *egfp-yscN*	this study
ADTM4520	IML421 *egfp-yscL*	this study
ADTM4525	IML421 *halo-yscL*	this study

Expression and suicide vectors

Plasmid	Relevant characteristics	References
pAD475	pBAD::*egfp* (*egfp* cloned in pBAD BglII/EcoRI sites)	this study
pAD476	pBAD::*mCherry* (*mCherry* cloned in pBAD BglII/EcoRI sites)	this study
pAD208	pKNG101 *yscV-egfp* (*egfp* and flexible linker cloned in-frame at the C-terminus of *yscV*)	this study
pAD447	pKNG101 *halo-yscQ* (*halo* and flexible linker cloned in-frame at the N-terminus of *yscQ*)	this study
pAD474	pKNG101 *egfp-yscK* (*egfp* and flexible linker cloned in-frame at the N-terminus of *yscK*)	this study
pADTM502	pKNG101 *egfp-yscN* (*egfp* and flexible linker cloned in-frame at the N-terminus of *yscN*)	this study
pADTM511	pKNG101 *egfp-yscL* (*egfp* and flexible linker cloned in-frame at the N-terminus of *yscL*)	this study
pADTM513	pKNG101 *halo-yscL* (*halo* and flexible linker cloned in-frame at the N-terminus of *yscL*)	this study
pBAD::*his*/B	pBR322-derived expression vector	Invitrogen
pKNG101	ori_{R6K} *sacBR*$^{+}$ $oriT_{RK2}$ *strAB*$^{+}$ (suicide vector)	[6]

Supplementary Table 3

Determination of expression ratio of soluble T3SS components under different conditions

The maximal intensity of 60 spots from three deconvoluted fields of view per strain in secreting and non-secreting conditions was corrected for the average background fluorescence in 30 unlabelled bacteria under the same conditions. To normalize for differences in exposure conditions and detection sensitivity, the ratio of the raw maximal fluorescence intensity under secreting and non-secreting conditions was normalized using a ratio of 2.45 for YscQ[71]. St.dev., standard deviation between fields of view.

	ratio secr./non-secr.		normalized ratio	
		st.dev.		st.dev.
**EGFP-YscQ**	0,1649	0,0257	**2,450**	0,381
**EGFP-YscK**	0,1463	0,0240	**2,173**	0,356
**EGFP-YscL**	0,1336	0,0336	**1,985**	0,498
**EGFP-YscN**	0,1326	0,0185	**1,970**	0,275

Supplementary Table 4

Exact number of data points

Exact number of data points, in case a range was given for n in the figure legend or main text

Dataset	Experiment	Strain / condition	Variables	
Fig. 1e + Table 1	automated detection of fluorescent foci		number of spots	number of bacteria
		wild-type	50	285
		EGFP-YscD	2244	262
		YscV-EGFP	1900	264
		EGFP-YscQ	3578	447
		EGFP-YscK	3640	537
		EGFP-YscL	3347	473
		EGFP-YscN	1968	274
Fig. 3a	FCS determination of transit time		number of analysed traces meeting quality criteria	
		EGFP	140	
		EGFP-YscK	73	
		EGFP-YscQ	199	
		EGFP-YscL	157	
		EGFP-YscN	86	
Fig. 3b	FCS determination of transit time		number of analysed traces meeting quality criteria	
		EGFP-YscK ΔYscQ	54	
		EGFP-YscQ ΔYscN	83	
		EGFP-$YscQ_{M218A}$	58	
		EGFP-YscL ΔYscN	65	
		EGFP-YscL ΔYscQ	79	
		EGFP-YscN ΔYscL	86	
Fig. 3c	FCS determination of		number of analysed traces	

	diffusion coefficient		meeting quality criteria
		EGFP-YscK, secreting	123
		EGFP-YscK, non-secreting	73
		EGFP-YscQ, secreting	142
		EGFP-YscQ, non-secreting	156
		EGFP-YscL, secreting	157
		EGFP-YscL, non-secreting	158
		EGFP-YscN, secreting	80
		EGFP-YscN, non-secreting	85
Fig. 3d, S11	FCS determination of transit time		number of analysed traces meeting quality criteria
		EGFP-YscQ, secreting (5 mM EDTA)	142
		EGFP-YscQ, no addition	65
		EGFP-YscQ, 1 mM $CaCl_2$	64
		EGFP-YscQ, non-secreting (5 mM $CaCl_2$)	156
		EGFP-YscL, secreting (5 mM EDTA)	157
		EGFP-YscL, no addition	67
		EGFP-YscL, 1 mM $CaCl_2$	84
		EGFP-YscL, non-secreting (5 mM $CaCl_2$)	158
Results, main text	FCS, comparison of conditions		number of analysed traces meeting quality criteria
		EGFP, secreting	79
		EGFP, non-secreting	39
Fig. S10de	FCS determination of transit time		number of analysed traces meeting quality criteria
		EGFP-YscL ΔYscD	29
		EGFP-YscL	16
		EGFP-YscQ ΔYscD	20
		EGFP-YscQ	13

Supplementary References:

1. Diepold, A., Kudryashev, M., Delalez, N. J., Berry, R. M. & Armitage, J. P. Composition, Formation, and Regulation of the Cytosolic C-ring, a Dynamic Component of the Type III Secretion Injectisome. *PLOS Biol.* **13,** e1002039 (2015).
2. Waithe, D., Clausen, M. P., Sezgin, E. & Eggeling, C. FoCuS-point: software for STED fluorescence correlation and time-gated single photon counting. *Bioinformatics* **32,** 958–60 (2016).
3. Clausen, M. P. *et al.* A straightforward approach for gated STED-FCS to investigate lipid membrane dynamics. *Methods* **88,** 67–75 (2015).
4. Hueck, C. J. Type III protein secretion systems in bacterial pathogens of animals and plants. *Microbiol. Mol. Biol. Rev.* **62,** 379–433 (1998).
5. Kudryashev, M. *et al.* In situ structural analysis of the Yersinia enterocolitica injectisome. *Elife* **2,** e00792 (2013).
6. Kaniga, K., Delor, I. & Cornelis, G. R. A wide-host-range suicide vector for improving reverse genetics in gram-negative bacteria: inactivation of the blaA gene of Yersinia enterocolitica. *Gene* **109,** 137–141 (1991).
